# Isolated Splenic Tuberculosis: A Diagnostic Conundrum

**DOI:** 10.7759/cureus.12958

**Published:** 2021-01-28

**Authors:** Sahil Grover, Yajur Arya, Saurabh Gaba, Monica Gupta, Arshi Syal

**Affiliations:** 1 Internal Medicine, Government Medical College & Hospital, Chandigarh, IND

**Keywords:** isolated splenic tuberculosis, pyrexia of unknown origin, spleen

## Abstract

Tuberculosis is an established cause of pyrexia of unknown origin and can implicate practically any human organ system. Splenic involvement is common in disseminated or miliary tuberculosis following hematogenous spread, but isolated splenic involvement is a very rare phenomenon. We report the case of a 30-year-old immunocompetent female who presented with high-grade fever and dull aching pain in the left hypochondrium for three months. Laboratory data provided no diagnostic information. Abdominal ultrasonography revealed an enlarged spleen with multiple small hypoechoic lesions that were corroborated on computed tomography. No pulmonary involvement or primary focus of infection was discernible elsewhere. Splenic fine needle aspiration cytology helped clinch a histopathological diagnosis of isolated splenic tuberculosis. Administration of anti-tubercular therapy resulted in resolution of the disease and an excellent outcome in our patient.

## Introduction

Although the spleen is the third most common organ affected in miliary or disseminated tuberculosis after the lungs and liver, isolated splenic tuberculosis is a rare disease manifestation [1]. The symptoms of splenic tuberculosis are usually non-specific and misleading. Fever, abdominal pain, and weight loss are common, although asymptomatic cases have also been reported [2]. Pyrexia of unknown origin (PUO) is a frequently reported disease manifestation [1]. Early diagnosis of isolated splenic tuberculosis is hardly possible given its vague symptomatology. In addition, *Mycobacterium tuberculosis* infection is challenging to diagnose in the spleen due to bacterial sequestration. Thus, a high degree of suspicion is necessary in cases presenting with PUO and splenomegaly.

## Case presentation

A 30-year-old female presented to the medicine department with complaints of fever and headache for three months, frequent bowel movements associated with abdominal pain, and generalized weakness for one month prior to presentation. Fever was insidious in onset, documented to be 101°F, and was associated with chills, rigors, and night sweats. The headache was diffuse in nature without any blurring of vision, vomiting, or neck stiffness. Her abdominal pain was localized to the left upper quadrant and was dragging in nature, non-radiating, and gradually progressive. It was associated with three to four loose stools a day which were watery in nature and not associated with blood or mucus. There was no history of cough, expectoration, loss of weight, or anorexia. There was no history of close contact with a patient of tuberculosis. Past history was significant for type 2 diabetes mellitus diagnosed three months previously and she was taking oral hypoglycemic agents. Her personal history did not suggest any addiction or allergies, exposure to irritants, intravenous or oral drug abuse, exposure to birds and pets, or human immunodeficiency virus (HIV) infection.

Physical examination revealed an average built female with a pulse rate of 100 beats per minute, respiratory rate of 14 per minute, blood pressure of 110/60 mmHg, and an axillary temperature of 100.4°F. On general physical examination, pallor was noted. There was no icterus, clubbing, lymphadenopathy, pedal edema, rash, scars, or sinuses. Abdominal examination revealed a non-tender, firm, enlarged spleen extending 4 cm below the costal margin. There was no evidence of hepatomegaly or free fluid in the abdomen. Respiratory system examination revealed bilaterally symmetrical air entry and normal vesicular breath sounds. Cardiovascular examination revealed normal heart sounds with no murmurs. Neck rigidity and other meningeal signs were absent. Ocular, ear, nose, and throat examinations were unremarkable. Gynecological examination was non-contributory. Examination of the spine did not reveal any spinal tenderness or palpable step-offs.

Initial laboratory investigations revealed a hemoglobin of 8.8 gm/dL, platelet count of 99 × 10^9^/L, a total leukocyte count of 3.9 × 10^9^/L (with normal differential counts), and erythrocyte sedimentation rate of 64 mm in the first hour. A peripheral blood smear was subsequently ordered which revealed a normocytic normochromic picture. The blood sugars were well-controlled, and her renal and liver function tests, serum electrolytes, and coagulation profile were within normal limits. C‑reactive protein was 90 mg/dL. Cultures of blood and urine were sterile. Serologic tests for hepatitis B, hepatitis C, and HIV were negative. Serum calcium, vitamin D, and angiotensin-converting enzyme levels were within normal range. Serological tests for other febrile tropical illnesses including dengue, malaria, scrub typhus, typhoid, brucellosis, and recombinant kinesin antigen (rK39) for leishmaniasis were negative. Chest radiography revealed no abnormalities. No acid-fast bacilli were detectable on sputum Ziehl-Neelsen (ZN) stain, and polymerase chain reaction (PCR) for *M. tuberculosis* was negative. The tuberculin skin test, however, showed an induration of 12 × 14 mm. It was considered positive as India is a high-risk country.

**Figure 1:**
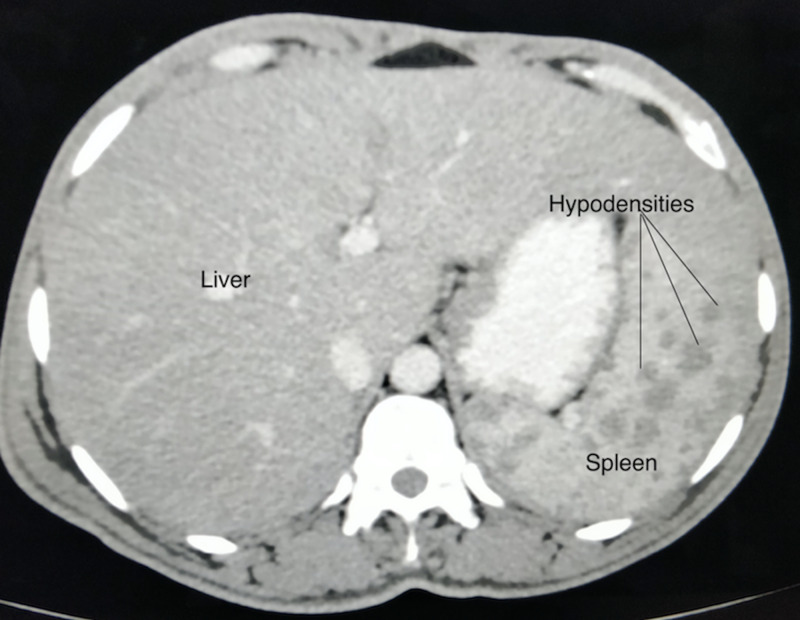
Abdominal CT scan showing splenomegaly with multiple hypoechoic lesions. CT, computed tomography

Ultrasonography of the abdomen was suggestive of splenomegaly measuring 16.6 cm, with multiple scattered well-defined hypoechoic lesions of variable sizes. Computed tomography (CT) scan of chest and abdomen revealed multiple well-defined scattered hypoechoic lesions of variable sizes in an enlarged spleen (Figure 1). The liver was normal with homogenous echogenicity. No mediastinal or abdominal lymphadenopathy was noted and the lung parenchyma was unremarkable.

The prominent differential possibilities in our case were lymphomatous infiltration, tuberculosis, sarcoidosis, and fungal infection in addition to multiple pyogenic abscesses.

**Figure 2:**
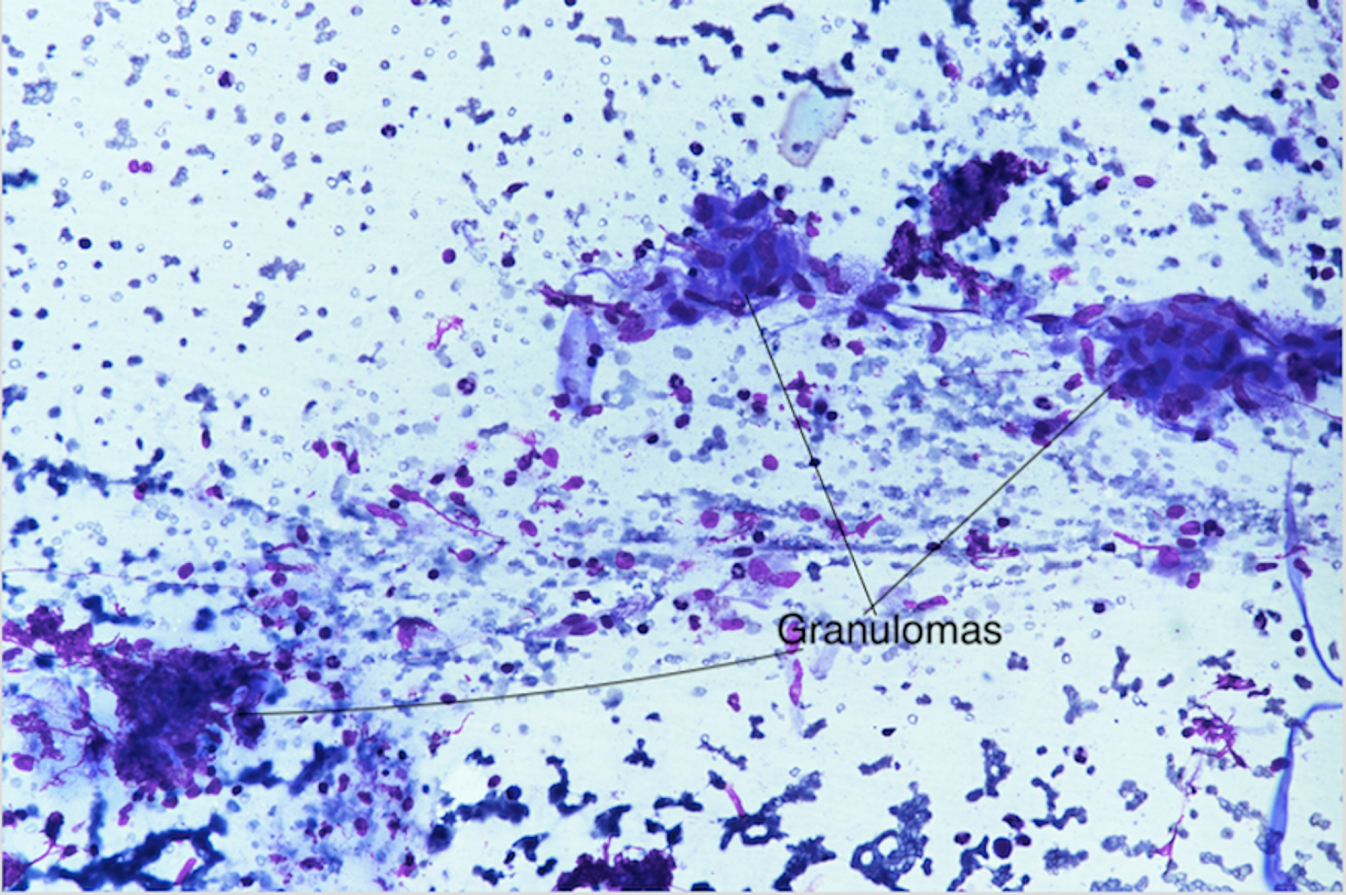
Splenic aspirate showing epithelioid cell granulomas.

**Figure 3:**
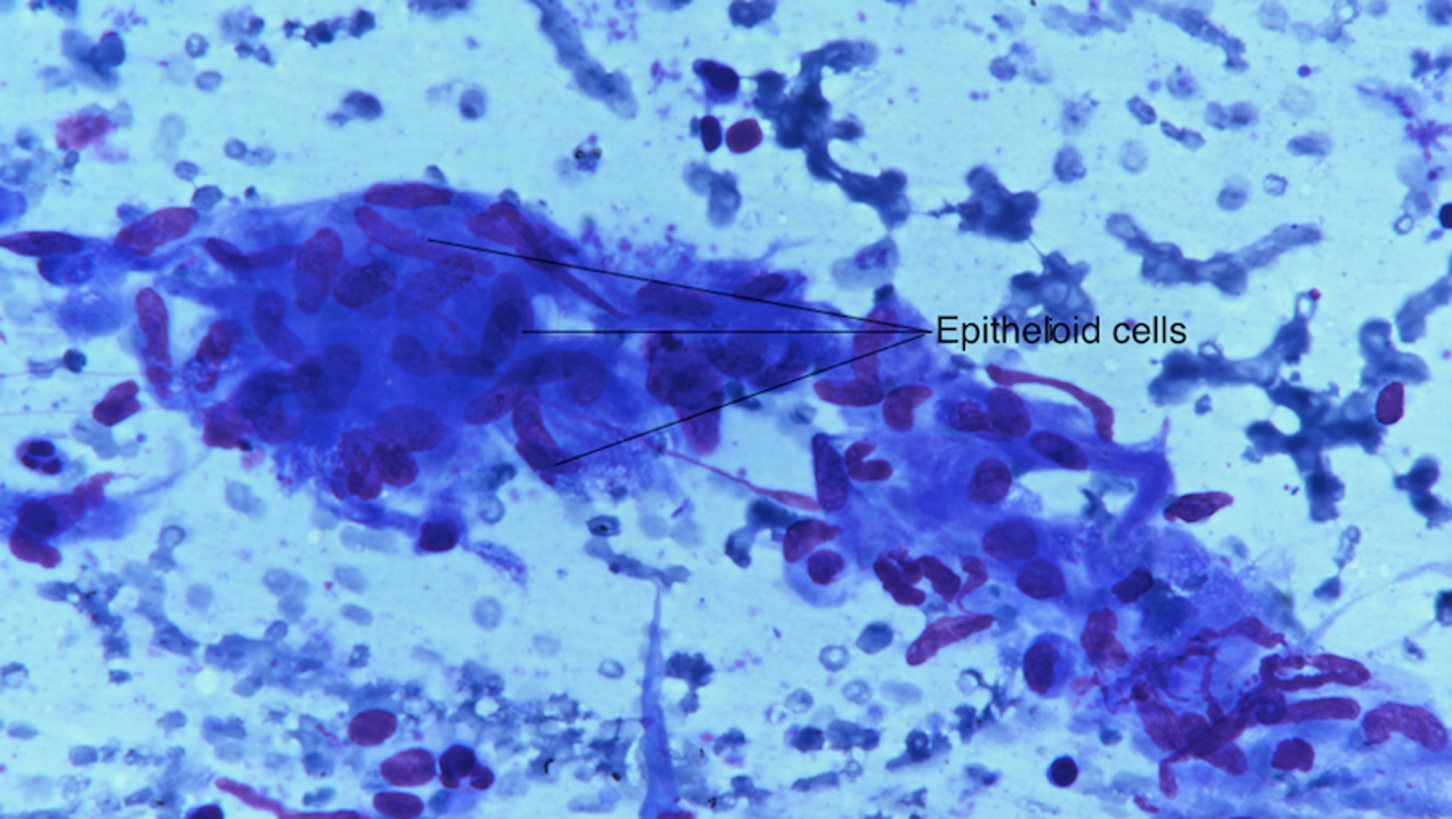
Splenic aspirate showing epithelioid cells.

A two-dimensional echocardiography was performed to rule out infective endocarditis and was found to be normal. Bone marrow aspiration and biopsy revealed mild megaloblastosis without any evidence of granulomas, acid-fast bacilli, or marrow infiltration. An ultrasound-guided fine needle aspiration cytology (FNAC) of the spleen was carried out, which revealed fragments of lymphoid and endothelial cells. A few epithelioid cell granulomas were also demonstrated (Figures 2, 3). Hence, a diagnosis of granulomatous inflammation was made.

No bacterial or fungal organisms were cultured from the aspirate. ZN staining for acid-fast bacilli of the aspirate was negative, however, PCR for *M. tuberculosis* of the aspirate was positive. The patient was started on standard quadruple anti-tubercular therapy (ATT) comprising isoniazid, rifampicin, pyrazinamide, and ethambutol for two months in the intensive phase followed by isoniazid and rifampicin for four months in the continuation phase. Her fever subsided by the end of two weeks. Repeat ultrasound at two months showed significant regression of the lesions as well as significant improvement in general well-being. ATT was administered for a total duration of six months.

## Discussion

Tuberculosis is a chronic infectious disease with a widespread prevalence in resource-poor countries. Although its incidence is on the decline in many developed nations, it is still a major public health problem in many developing countries. Primary splenic (isolated) tuberculosis is now a recognized entity, although it is extremely rare, especially in immunocompetent patients. Therefore, one must exclude immunosuppression, repeated pyogenic infections, prior splenic trauma or splenic defects, and hemoglobinopathies such as sickle cell disease [3]. To date, only a limited number of case reports have been published related to isolated splenic tuberculosis, as described below (Table 1).

**Table 1:** Case reports on isolated splenic tuberculosis in the last decade. M, male; F, female; USG, ultrasound; ZN, Ziehl-Neelsen; ATT, anti-tubercular therapy; PUO, pyrexia of unknown origin; CNB, core needle biopsy; LUQ, left upper quadrant; FNAC, fine needle aspiration cytology; FDG-PET, fluorodeoxyglucose-positron emission tomography; CT, computed tomography

S. No.	Authors	Patient details (Age/Gender)	Presenting symptoms/signs	Diagnosis	Management
1.	Udgaonkar et al. [2]	38/F	Asymptomatic and incidentally diagnosed on USG abdomen	ZN staining on USG-guided aspiration of the spleen	Six months of ATT
2.	Basa et al. [3]	27/M	Generalized weakness, anorexia, weight loss, night sweats, splenomegaly, and thrombocytopenia	Histopathology of splenectomy specimen	Splenectomy followed by ATT
3.	Wangai et al. [4]	26/M	PUO	CNB of the spleen	Six months of ATT
4.	Gupta et al. [5]	62/F	Low-grade fever and LUQ pain	USG-guided FNAC of the spleen	Six months of ATT
5.	Dhibar et al. [6]	30/M	LUQ pain, weight loss and splenomegaly	USG-guided FNAC of the spleen	Six months of ATT
6.	Rao et al. [7]	17/F	PUO	FDG-PET and Trucut biopsy of the spleen	Nine months of ATT
7.	Kumar et al. [8]	23/F	PUO and LUQ pain	Histopathology of splenectomy specimen	Splenectomy followed by six months of ATT
8.	Mandal et al. [9]	20/F	PUO, weight loss, and splenomegaly	USG-guided FNAC of spleen	Nine months of ATT
9.	Mishra et al. [10]	28/M	LUQ pain	Frozen section of the spleen during laparotomy	Splenectomy followed by ATT
10.	Zhan et al. [11]	36/M	PUO with splenomegaly	CNB of the spleen	Six months of ATT
11.	Kumar et al. [12]	51/M	PUO and LUQ pain with splenomegaly	CT-guided FNAC of the spleen	Six months of ATT
12.	Kundu et al. [13]	13/M	LUQ pain with splenomegaly	FNAC of the spleen	Splenectomy followed by eight months of ATT

Splenic tuberculosis can present with a myriad of signs and symptoms ranging from constitutional symptoms, such as fatigue, weight loss, splenomegaly, or PUO, to grave complications including hypersplenism, portal hypertension, and splenic rupture [4]. Generally, splenic tuberculosis is associated with reduced cell counts, as seen in our patient; however, polycythemia has also been documented. The initial imaging modality of choice is abdominal ultrasound as it is cost-effective and helps in characterizing the lesion. Splenic lesions typically present as multiple, hypoechoic lesions which may be tuberculomas or abscesses. Abdominal CT scan has higher diagnostic accuracy and can aid in superior delineation of splenic lesions. Diagnosis is further clinched by histopathological examination of the lesions. In various reports (Table 1), either FNAC, laparoscopic, or imaging-guided core needle biopsy (CNB) or splenectomy specimens have been examined for diagnostic confirmation [14]. CNB has a higher diagnostic yield and superior accuracy compared to FNAC in characterizing splenic lesions [15]. FNAC has a lower risk of bleeding compared to CNB.

ATT forms the mainstay of management in splenic tuberculosis. Duration of treatment varies but regimens generally lasting for six to nine months are deemed satisfactory. In patients who do not respond to medical therapy alone, splenectomy forms a viable option. A paradoxical phenomenon of exaggerated inflammatory symptoms, called immune reconstitution inflammatory syndrome, can rarely be seen on initiation of medical therapy. This may present as either worsening of existing lesions or development of new lesions, or as worsening of clinical or radiological findings following initiation of ATT [16].

## Conclusions

Isolated splenic tuberculosis is a rare presentation of tuberculosis and has been occasionally described in the literature. Common symptoms include fever, abdominal pain, diarrhea, and generalized weakness. Significant diagnostic delays usually occur due to its inconsistent symptomatology. Ultrasound and CT scan of the abdomen are the first-line investigations. Tissue aspiration or biopsy are indispensable for diagnostic confirmation in cases where splenectomy is not performed. Amplification modalities including PCR techniques are useful for establishing diagnosis in ZN stain-negative cases. Administration of ATT can result in a favorable outcome and can preclude the need of a splenectomy. Physicians should be cognizant of isolated splenic affliction in tuberculosis, and tissue diagnosis should be carried out as early as possible in suspected cases.
